# Platelet Supernatant Suppresses LPS-Induced Nitric Oxide Production from Macrophages Accompanied by Inhibition of NF-κB Signaling and Increased Arginase-1 Expression

**DOI:** 10.1371/journal.pone.0162208

**Published:** 2016-09-02

**Authors:** Yusuke Ando, Teruaki Oku, Tsutomu Tsuji

**Affiliations:** Department of Microbiology, Hoshi University School of Pharmacy and Pharmaceutical Sciences, Tokyo, Japan; Fundacao Oswaldo Cruz, BRAZIL

## Abstract

We previously reported that mouse bone marrow-derived macrophages (BMDMs) that had been co-cultured with platelets exhibited lower susceptibility to bacterial lipopolysaccharide (LPS) and produced lower levels of nitric oxide (NO) and inflammatory cytokines including TNF-α and IL-6. The suppression of macrophage responses was mediated, at least in part, by platelet supernatant. In the present study, we assessed phenotypic changes of BMDMs induced by incubation with the supernatant from thrombin-activated platelets (PLT-sup) and found that BMDMs cultured with PLT-sup (PLT-BMDMs) expressed a lower level of inducible NO synthase (iNOS) and a higher level of arginase-1, both of which are involved in the L-arginine metabolism, upon stimulation with LPS or zymosan. We also examined possible modulation of the NF-κB signaling pathway and observed suppression of IκBα phosphorylation and a decrease of NF-κB p65 expression in LPS-stimulated PLT-BMDMs. These results suggest that PLT-sup suppresses inflammatory responses of BMDMs via negative regulation of NF-κB signaling leading to lowered expression of iNOS and enhanced L-arginine catabolism by arginase-1.

## Introduction

Bacterial lipopolysaccharide (LPS), a major component of the outer membrane of Gram-negative bacteria, stimulates macrophages to produce various inflammatory cytokines, including tumor necrosis factor-α (TNF-α), interleukin-1 (IL-1), and IL-6 [1–3]. In addition to these cytokines, nitric oxide (NO) produced by activated macrophages plays important roles in the pathogenesis of inflammation related to bacterial infection [4–7]. Excessive macrophage activation with release of these inflammatory mediators causes systemic inflammatory response syndrome (SIRS), disseminated intravascular coagulation (DIC), and multiple organ failure (MOF). We recently reported that bone marrow-derived macrophages (BMDMs) that had been co-cultured with platelets exhibited lower susceptibility to LPS and produced lower levels of NO, TNF-α, and IL-6 [8]. The suppression of macrophage responses by platelets did not necessarily require the direct cell—cell contact between macrophages and platelets, but appeared to be mediated by soluble factors secreted from platelets upon stimulation with thrombin or other stimulants. These observations provided a cellular basis for the results of an earlier *in vivo* study showing that thrombocytopenia increased mortality and aggravated organ failure in LPS-induced endotoxemia [9] and also supported the notion that platelets play critical roles in the modulation of inflammatory responses.

Nitric oxide produced by activated macrophages is thought to be responsible for bacterial killing, tumor cytotoxicity and virus inactivation [10–12]. Moreover, NO interacts with reactive oxygen species (ROS) to generate peroxynitrite, which is a powerful oxidizing agent [13]. However, the excessive production of NO causes tissue damage, extensive systemic vasodilatation and hypotension, leading to organ hypoperfusion and dysfunction in association with septic shock [14–18]. The biosynthesis of NO is catalyzed by the enzyme nitric oxide synthase (NOS), which converts L-arginine into L-citrulline and NO. Three isoforms of NOS, neuronal NOS (nNOS, NOS1), inducible NOS (iNOS, NOS2), and endothelial NOS (eNOS, NOS3), are involved in the NO synthesis. The expression of nNOS and eNOS are constitutive in the brain and endothelium, respectively, whereas iNOS is expressed in response to cytokines and bacterial components, including LPS. Arginase is another enzyme involved in the L-arginine metabolism and converts L-arginine into L-ornithine and urea. Two isoforms of arginase, arginase-1 and arginase-2, exist in mammals and differ in their tissue distribution and physiologic functions [19, 20]. Arginase-1 is constitutively expressed in the liver as one of the enzymes of the urea cycle, whereas its expression in macrophages is regulated by Th2 cytokines such as IL-4 and IL-13 [21]. The macrophage arginase-1 expression suppresses NO production by competing with iNOS for L-arginine [19, 22–24]. Thus, the iNOS/arginase-1 balance in macrophages affects inflammatory responses through the L-arginine metabolism. The expressions of iNOS and arginase-1 are also important for the functional classification of macrophages [23, 25], M1-macrophages (also referred to as classically activated macrophages: CAMs) and M2-macrophages (alternatively activated macrophages: AAMs). M1-macrophages are characterized by the expression of TNF-α, IL-1, IL-6 and iNOS upon stimulation with various bacterial components and Th1 cytokines [26], whereas M2-macrophages exhibit anti-inflammatory phenotypes including the expressions of IL-10, transforming growth factor-β (TGF-β) and arginase-1, and play roles in immune tolerance, tissue repair, wound healing and fibrosis [27].

In the present study, we characterized macrophage phenotypic changes induced by the supernatant from activated platelets and found that the expression upon stimulation with bacterial components (LPS and zymosan) of two enzymes involved in the L-arginine metabolism, iNOS and arginase-1, was influenced by platelet-derived factors. The results also suggested that modulation of the NF-κB signaling pathway was involved in the process.

## Materials and Methods

### Reagents, antibodies, and cell culture

Lipopolysaccharide (LPS) (*Escherichia coli* O-111), MEM non-essential amino acids solution (x100), 2-mercaptoethanol (2-ME), and kanamycin sulfate were purchased from Wako Pure Chemical Industries (Osaka, Japan). Zymosan (*Saccharomyces cerevisiae*), thrombin, Triton X-100, α-isonitrosopropiophenone, and a protease inhibitor cocktail were from Sigma-Aldrich (St. Louis, MO, USA). RPMI 1640 medium and ASF104 serum-free medium were products of Sigma-Aldrich and Ajinomoto (Tokyo, Japan), respectively. Fetal bovine serum (FBS) was purchased from Biosera (Boussens, France). Oligonucleotides were supplied by FASMAC Co., Ltd. (Kanagawa, Japan).

Anti-mouse TLR4/MD-2 complex antibody (MTS510) and anti-mouse CD16/32 antibody (93) were purchased from Affymetrix (Santa Clara, CA, USA). Biotin-conjugated anti-mouse CD14 antibody (Sa14-2) and streptavidin-PE were products of Biolegend (San Diego, CA, USA). Anti-mouse iNOS antibody (54/iNOS) was from BD Biosciences (Franklin Lakes, NJ, USA). Anti-Arginase-1 antibody (EPR6671 (B)) was a product of Abcam (Cambridge Science Park, Cambridge, UK). Anti-GAPDH antibody (6C5) was purchased from EMD Millipore (Billerica, MA, USA). Antibodies against IκBα (L35A5), phospho-IκBα (Ser32) (14D4), NF-κB p65 (D14E12), phospho-NF-κB p65 (Ser536) (93H1), p38 MAPK (D13E1), phospho-p38 MAPK (Thr180/Tyr182) (D3F9), SAPK/JNK (#9252), phospho-SAPK/JNK (Thr183/Tyr185) (81E11), ERK1/2 (137F5) and phospho-ERK1/2 (Thr202/Tyr204) (D13.14.4E), goat anti-rabbit IgG HRP-linked antibody, and horse anti-mouse IgG HRP-linked antibody were products of Cell Signaling Technology, Inc. (Danvers, MA, USA). Anti-histone H3 antibody (MABI0301) was purchased from MBL (Nagoya, Japan). Goat anti-rat IgG-Alexa Fluor 647 antibody was from Thermo Fisher Scientific (Waltham, MA, USA).

L929 cells were supplied by Riken Cell Bank (Tsukuba, Japan) and cultured with RPMI 1640 medium supplemented with 10% heat-inactivated FBS, non-essential amino acids, 2-ME (50 μM) and kanamycin sulfate (100 μg/mL) (complete medium) at 37°C under humidified 5% CO_2_. The conditioned medium was obtained by centrifugation (2,000 x g, 20 min) followed by filtration with a membrane (0.22 μm pore).

### Mice

BALB/c mice at 5–8 weeks of age were supplied by Japan SLC, Inc. (Shizuoka, Japan). All procedures for experiments using mice were conducted in accordance with the Guide for Care and Use of Laboratory Animals of Hoshi University School of Pharmacy and Pharmaceutical Sciences. The animal protocols were approved by the Animal Care and Use Committee of Hoshi University School of Pharmacy and Pharmaceutical Sciences (protocol: #27–055). All experiments were performed under diethyl ether anesthesia, and every effort was made to minimize animal suffering and reduce the number of animals used.

### Bone marrow-derived macrophages (BMDMs)

Mouse BMDMs were prepared as previously described [8]. Briefly, bone marrow cells were isolated from BALB/c mice and cultured for 6 days in complete medium containing L929 conditioned medium (10%). The adherent cells were washed with serum-free RPMI 1640 medium and cultured in complete medium without L929 conditioned medium for 24 h.

### Macrophage culture with platelet supernatant

Platelets were prepared from mouse blood collected by cardiac puncture as previously described [8]. Purity of the platelet preparation was >99% (CD41^+^) as estimated by flow cytometry. Washed platelets were suspended in ASF104 serum-free medium (1 × 10^8^ cells/mL) and stimulated with thrombin (0.5 U/mL) for 15 min at 37°C. Flow cytometric analysis using anti-CD62P (P-selectin) antibody confirmed that >95% of platelets were activated to express CD62P on the cell surface (S1 Fig). The supernatant was collected by centrifugation (800 × g, 15 min, 4°C) and filtered with a membrane (0.22 μm pore) (PLT-sup).

BMDMs were cultured with PLT-sup at 37°C under humidified atmosphere (5% CO_2_) for 24 h (PLT-BMDMs). The culture of BMDMs with thrombin (0.5 U/mL) alone was also conducted as a control (control BMDMs).

### Measurement of nitrite

The NO production was determined by measuring NO_2_^-^ concentrations in the culture supernatant by Griess reaction as previously described [8]. Briefly, cell-free BMDM culture supernatant was mixed with an equal volume of Griess reagent (1% sulfanilamide, 0.1% *N*-(1-naphthyl)ethylendiamine dihydrochloride, 2.5% phosphoric acid) in a 96-well plate and incubated at room temperature for 10 min. Absorbance at 570 nm was then measured with a microplate reader (MTP-450; Corona Electric Co., Ltd., Ibaraki, Japan).

### Arginase assay

Arginase activity was measured as previously described with slight modifications [28]. Cells (4 × 10^5^ cells) were lysed in 0.1% Triton X-100 (100 μL) containing a protease inhibitor cocktail for 10 min at room temperature. After the cell lysate was obtained by centrifugation at 20,000 × g for 20 min at 4°C, an aliquot (18 μL) was mixed with 0.5 M Tris-HCl (pH 7.5) (1 μL) and 0.1 M MnCl_2_ (1 μL) and incubated at 56°C for 10 min. The hydrolysis reaction of L-arginine was then performed by incubating the cell lysate (20 μL) with 0.5 M L-arginine (pH 9.7) (20 μL) at 37°C for 60 min and terminated by adding a stop solution (H_2_SO_4_/H_3_PO_4_/H_2_O, 1:3:7) (160 μL). The concentration of urea liberated was determined colorimetrically by the addition of 9% α-isonitrosopropiophenone in ethanol (8 μL), followed by heating at 100°C for 45 min. After placing the plate in the dark for 10 min at room temperature, the absorbance at 570 nm was measured with a microplate reader, and arginase activity was normalized by the protein concentration in the cell lysate, which was quantified using a BCA Protein Assay Kit (Thermo Fisher Scientific).

### RT-qPCR

Total RNA was extracted from cells using TRIzol reagent (Life Technologies, Carlsbad, CA, USA), and cDNA was synthesized from the total RNA using a PrimeScript RT reagent Kit (TaKaRa, Shiga, Japan). The procedures were performed according to the respective manufacturer’s protocols. The qPCR reactions were conducted in an Applied Biosystems StepOne system (Life Technologies) with KAPA SYBR FAST qPCR Kit Master Mix (2X) ABI Prism (KAPA Biosystems, Boston, MA, USA). All samples were analyzed in triplicate and quantified by the relative standard curve method using the *Gapdh* housekeeping gene as an internal control. The sequences of the primers used are listed in Table 1.

**Table 1:** Oligodeoxynucleotide primers used in qPCR experiments.

Gene	Forward (5’-3’)	Reverse (5’-3’)
*Gapdh*	TGAAGCAGGCATCTGAGGG	CGAAGGTGGAAGAGTGGGAG
*Tnf*	CAGGCGGTGCCTATGTCTC	ATGAGAGGGAGGCCATTTGG
*Il6*	ATACCACTCCCAACAGACCTGTC	TTTCTGCAAGTGCATCATCGTTG
*Il1b*	CAACCAACAAGTGATATTCTCCATG	GATCCACACTCTCCAGCTGCA
*Nos2*	TCCAGGGATTCTGGAACATT	GAAGAAAACCCCTTGTGCTG
*Arg1*	CATGAGCTCCAAGCCAAAGT	TTTTTCCAGCAGACCAGCTT
*Fizz1*	ACTGCCTGTGCTTACTCGTTGACT	AAAGCTGGGTTCTCCACCTCTTCA
*Ym1*	CACCATGGCCAAGCTCATTCTTGT	TATTGGCCTGTCCTTAGCCCAACT
*Mrc1*	CCACAGCATTGAGGAGTTTG	ACAGCTCATCATTTGGCTCA

### Western blotting

BMDMs were lysed in 1 × SDS sample buffer (50 mM Tris-HCl, 1% SDS, 5% glycerol, 0.01% bromophenol blue, pH 6.8). The resultant cell lysates were sonicated and subjected to SDS-polyacrylamide gel electrophoresis (PAGE). SDS-PAGE and western blotting were conducted as described previously [29].

### Cell Fractionation

BMDMs (2.5 × 10^6^ cells) cultured in a 3.5 cm dish were incubated with a hypotonic buffer (20 mM Tris-HCl, 10 mM NaCl, 3 mM MgCl_2_, pH 7.4) (200 μL) containing 0.5% Nonidet P-40 on ice for 15 min. The cells were then recovered with a scraper into a test tube, and the tube was agitated vigorously for 10 sec with a vortex mixer. The mixture was separated by centrifugation at 1,000 × g for 10 min into a supernatant (cytoplasmic fraction) and pellet (nuclear fraction).

### Data analysis

Statistical data analysis was conducted using the Mann-Whitney U-test, and significance was considered at *p* < 0.05.

## Results

### The supernatant of activated platelets suppressed LPS-induced macrophage NO production

**Fig 1:**
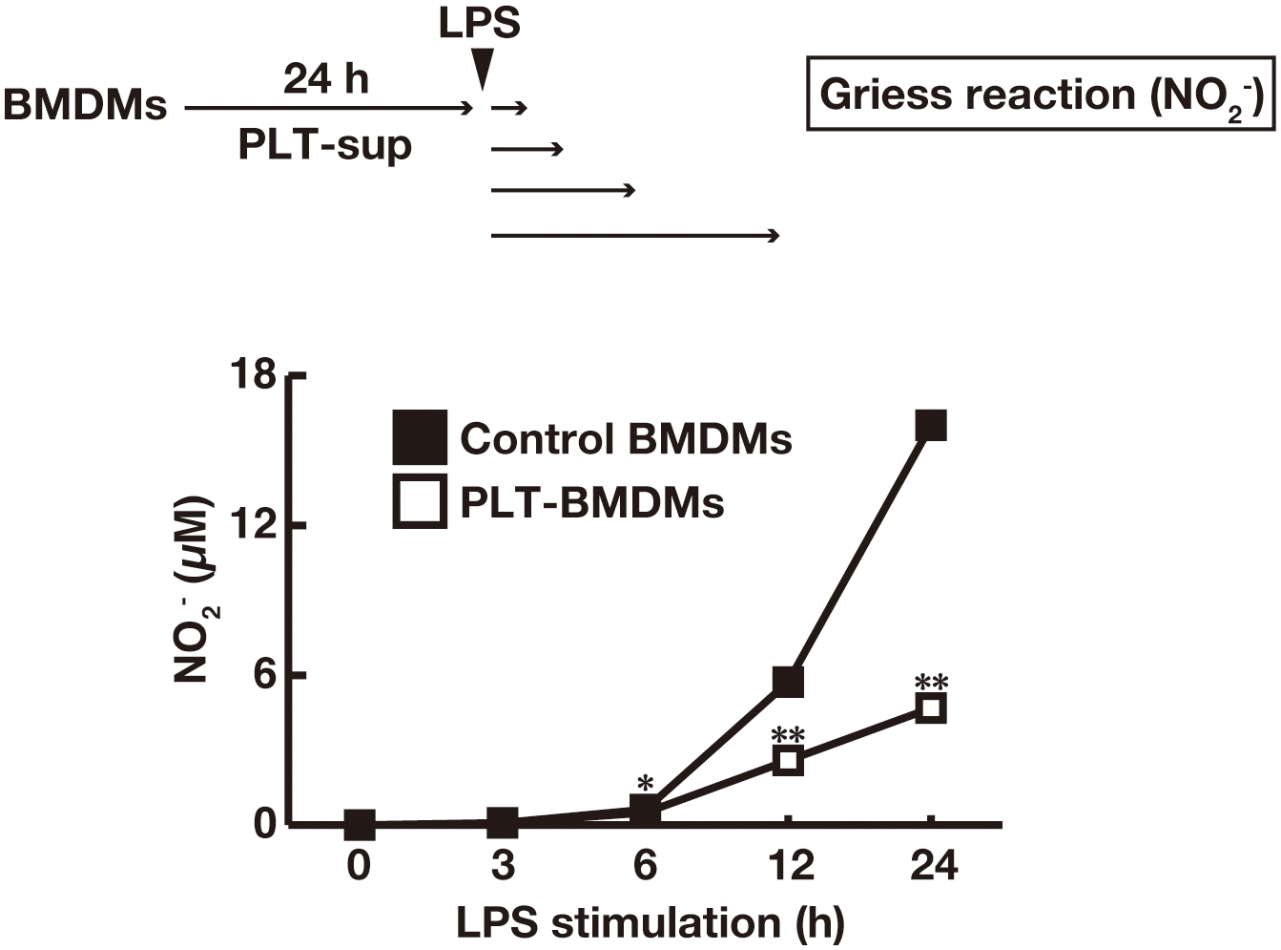
Attenuation of LPS-induced NO production from BMDMs by PLT-sup. BMDMs (2.5 × 10^6^ cells) were cultured for 24 h with PLT-sup (PLT-BMDMs) or 0.5 U/mL thrombin alone (control BMDMs) in a 3.5 cm dish, and stimulated with complete medium containing LPS (50 ng/mL) for 0–24 h. The production levels of NO_2_^-^ in the culture supernatant were determined by Griess reaction using a calibration curve for NaNO_2_. Experiments were performed in quintuplicate and repeated four times. Data are presented as the mean ± SEM. *p < 0.05, **p < 0.01 vs. control BMDMs. Representative results of the four experiments are shown.

We first conducted a time-course experiment of the effect of thrombin-stimulated platelet supernatant (PLT-sup) on the LPS-induced NO production by macrophages. When BMDMs were cultured with PLT-sup for 24 h (PLT-BMDMs) and stimulated with LPS for various time periods (0–24 h), PLT-BMDMs were found to produce a lower level of NO after 12–24 h stimulation with LPS as compared to control BMDMs (Fig 1). Similar results were observed when BMDMs were cultured with supernatants of ADP- or collagen-stimulated platelets (S2 Fig). These results suggest that phenotypic changes of BMDMs may take place to attenuate their susceptibility to LPS in the presence of soluble factors derived from activated platelets.

**Fig 2:**
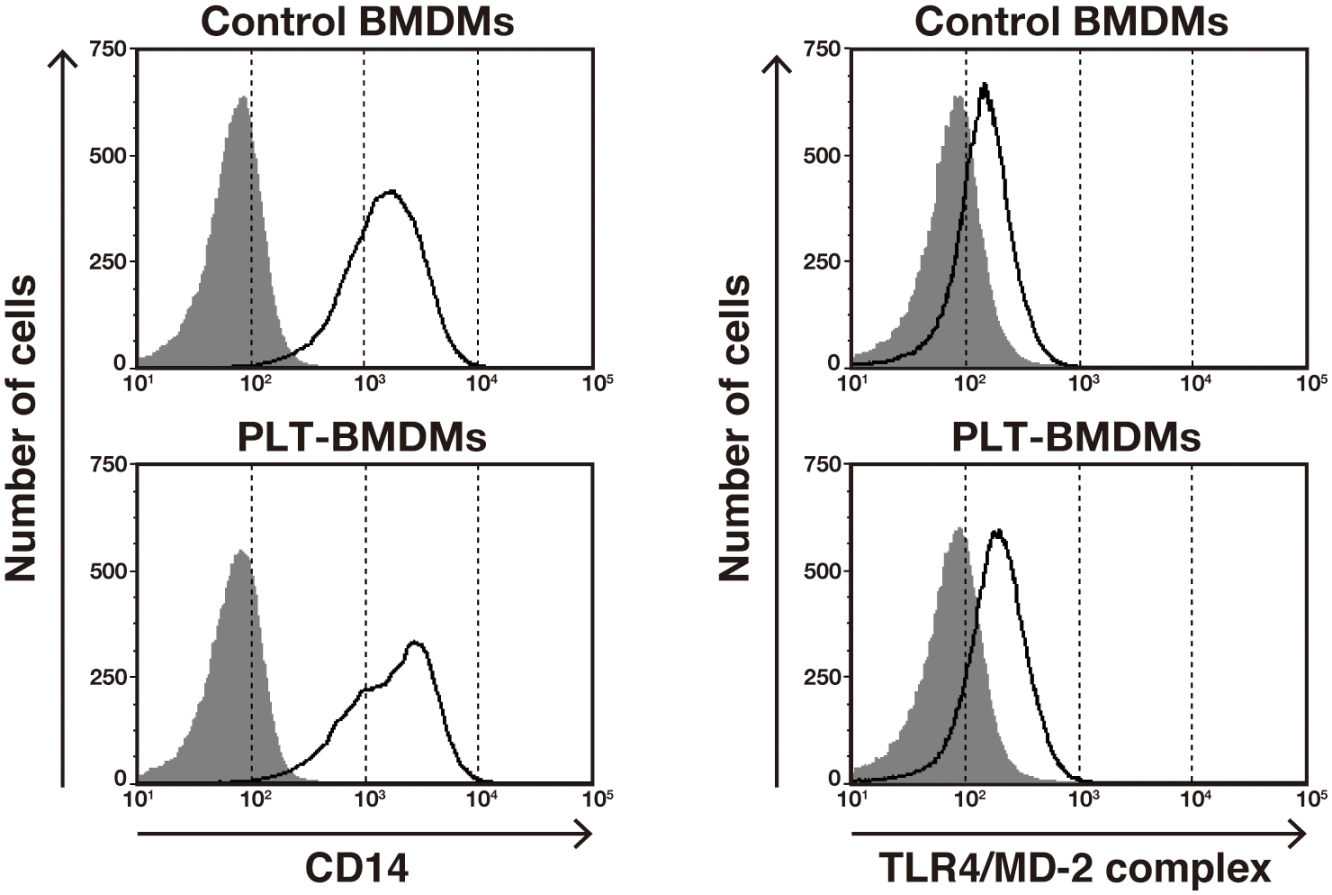
Cell surface expression of CD14 and the TLR4/MD-2 complex in PLT-BMDMs. PLT-BMDMs and control BMDMs were incubated with anti-mouse CD16/32 at 4°C for 10 min, and then incubated with biotin anti-mouse CD14 antibody or rat anti-mouse TLR4/MD-2 complex antibody at 4°C for 30 min. After incubation with streptavidin-PE or anti-rat IgG-Alexa Fluor 647 antibody at 4°C for 30 min, cells were analyzed by FACSVerse (BD Biosciences). The data are shown as histograms representing the number of cells (Y-axis) against the log of fluorescence intensity (X-axis). The black lines represent cells with primary antibody, and the gray-shaded areas represent cells without primary antibody. Experiments were repeated three times, and representative results are shown.

We next examined possible changes in the surface expression of LPS receptors on BMDMs after the culture with PLT-sup. LPS is known to bind to LPS-binding protein (LBP), and the resulting complex is recognized by cell surface receptor CD14. The LPS-LBP-CD14 conjugate is then transferred to the complex of Toll-like receptor 4 (TLR4) and myeloid differentiation factor 2 (MD-2), leading to the generation of an intracellular activation signal [30]. We therefore analyzed the expression levels of CD14 and the TLR4/MD-2 complex on the PLT-BMDM cell surface by flow cytometry. As shown in Fig 2, no significant changes in the expression of CD14 and the TLR4/MD-2 complex were observed between PLT-BMDMs and control BMDMs. It is therefore unlikely that the attenuation of LPS-induced NO production in PLT-BMDMs was due to a decrease in the expression of CD14 or the TLR4/MD-2 complex.

### PLT-sup modulated expression of arginase-1 and iNOS in macrophages

**Fig 3:**
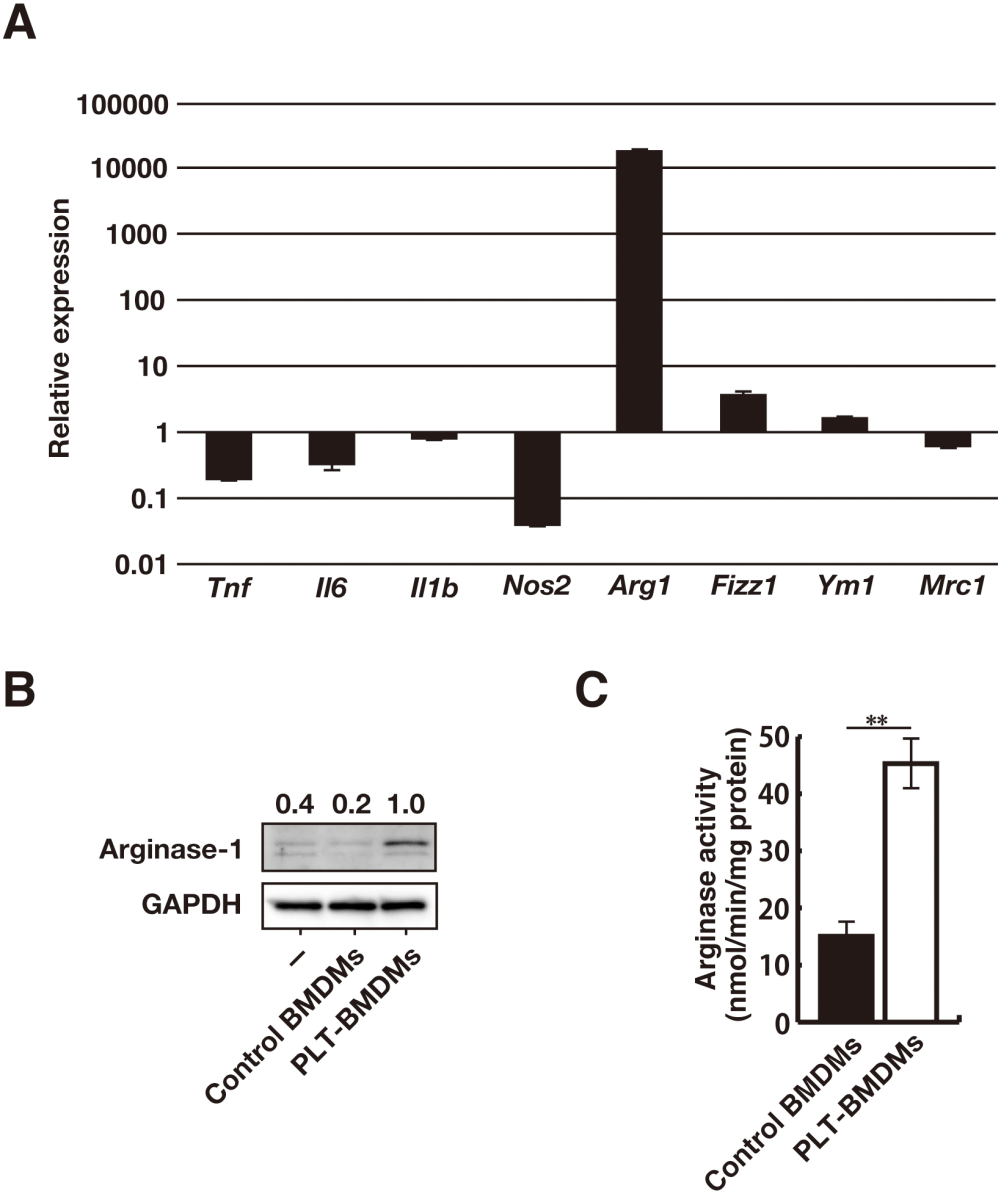
Induction of arginase-1 expression by PLT-sup in BMDMs. (A) The gene expressions of *Tnf*, *Il6*, *Il1b*, *Nos2*, *Arg1*, *Fizz1*, *Ym1*, and *Mrc1* in PLT-BMDMs and control BMDMs were analyzed by RT-qPCR with the relative standard curve method using *Gapdh* as an internal control. The gene expression in PLT-BMDMs was represented as the value relative to gene expression in control BMDMs. Experiments were performed in triplicate and repeated three times. Data are presented as the mean ± SEM, and representative results of the three experiments are shown. (B) PLT-BMDMs and control BMDMs were lysed in 1 × SDS sample buffer, and the resultant lysates were subjected to western blotting analysis with antibodies against arginase-1 or GAPDH. The relative intensity of each arginase-1 band after normalization to the corresponding levels of GAPDH is shown above each blot. Experiments were repeated five times, and representative results are shown. (C) PLT-BMDMs and control BMDMs (4 × 10^5^ cells) were lysed with 0.1% Triton X-100 (100 μL) containing a protease inhibitor cocktail for 10 min at room temperature, and the lysate was assayed for arginase activity as described in the Materials and Methods. Experiments were performed in quintuplicate and repeated five times. Data are presented as the mean ± SEM. **p < 0.01 vs. control BMDMs. Representative results of the five experiments are shown.

We then assessed the gene expression levels for known macrophage polarization markers. For RT-qPCR analysis, we selected four M1-macrophage markers (*Tnf*, *Il6*, *Il1b*, *Nos2*) and four M2-macrophage markers (*Arg1*, *Fizz1*, *Ym1*, *Mrc1*) and compared the mRNA levels in PLT-BMDMs with those in control BMDMs. As shown in Fig 3A, we found dramatic changes in the mRNA levels of two enzymes involved in L-arginine metabolism—i.e., an increase of *Arg1* (ariginase-1) expression and a decrease of *Nos2* (iNOS) expression in PLT-BMDMs. However, the other macrophage polarization markers (*Tnf*, *Il6*, *Il1b*, *Fizz1*, *Ym1*, and *Mrc1*) exhibited no significant changes. These results indicate that PLT-sup induces M2-like polarization of BMDMs, but the resulting macrophages are different from typical M2-macrophages. The induction of arginase-1 expression in BMDMs by PLT-sup was also confirmed at a protein level. The western blotting analysis (Fig 3B) and the arginase activity assay (Fig 3C) revealed that PLT-BMDMs expressed a higher level of arginase-1 than control BMDMs.

**Fig 4:**
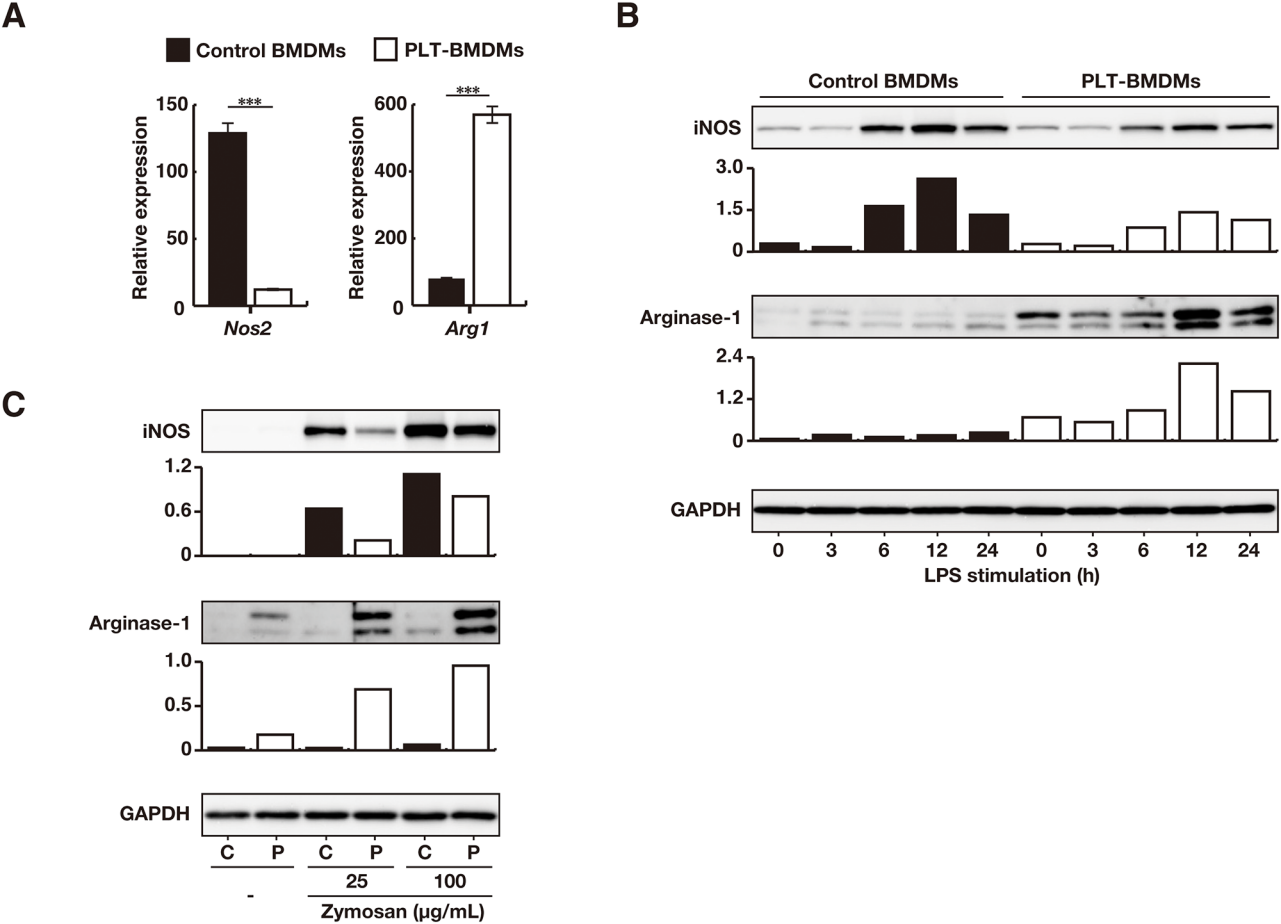
Expression of iNOS and arginase-1 in BMDMs after LPS stimulation. (A) PLT-BMDMs and control BMDMs (2.5 × 10^6^ cells) were stimulated with LPS (50 ng/mL) for 24 h, and the gene expressions of *Nos2* and *Arg1* were analyzed by RT-qPCR with the relative standard curve method using *Gapdh* as an internal control. The gene expression is represented as the value relative to gene expression in the original BMDMs. Experiments were performed in quintuplicate and repeated four times. The data are presented as the mean ± SEM. ***p < 0.005 vs. control BMDMs. Representative results of the four experiments are shown. (B) PLT-BMDMs and control BMDMs (2.5 × 10^6^ cells) were stimulated with LPS (50 ng/mL) for 0–24 h. Cells were then lysed in 1 × SDS sample buffer, and the cell lysates were subjected to western blotting analysis with antibodies against iNOS, arginase-1 or GAPDH. The relative intensity of each iNOS or arginase-1 band after normalization to the levels for GAPDH is shown in the lower panel. Experiments were repeated four times, and representative results are shown. (C) PLT-BMDMs and control BMDMs (2.5 × 10^6^ cells) were stimulated with zymosan (25 or 100 μg/mL) for 12 h, and then cell lysates were subjected to western blotting analysis with antibodies against iNOS, arginase-1 or GAPDH. *C*, control BMDMs; *P*, PLT-BMDMs. The relative intensity of each iNOS or arginase-1 band after normalization to the levels for GAPDH is shown in the lower panel. Experiments were repeated four times, and representative results are shown.

The importance of the iNOS/arginase-1 balance for NO production by macrophages has recently been discussed [23]. Since intracellular arginase-1 expression was reported to be influenced by TLR-mediated macrophage activation [31, 32], we analyzed changes in the mRNA expression of *Nos2* and *Arg1* after LPS stimulation by RT-qPCR. When PLT-BMDMs and control BMDMs were stimulated with LPS for 24 h, the expression level of *Arg1* in PLT-BMDMs was found to be 7-fold higher than that in control BMDMs, in association with lower (~10%) expression of *Nos2* (Fig 4A). We then assessed the protein level expression of iNOS and arginase-1 by western blotting after various time periods of LPS stimulation. The iNOS expression in control BMDMs was found to be markedly increased 6 h after the LPS stimulation and maintained at higher levels up to 24 h, whereas the expression in PLT-BMDMs appeared to be suppressed at 6–24 h after the LPS stimulation (Fig 4B). By contrast, the expression of arginase-1 (p35 and p38) in PLT-BMDMs was greatly increased upon LPS stimulation. Based on these results, both lower expression of iNOS and higher expression of arginase-1 in PLT-BMDMs were considered likely to contribute to the attenuation of NO production by these cells. Indeed, when the LPS-induced NO production from PLT-BMDMs in the presence of an arginase-1 inhibitor (nor-NOHA) was measured, a partial restoration of NO production was observed (S3 Fig). Thus arginase-1 was suggested to play a slight but significant role in the reduced NO production by PLT-BMDM through the L-arginine catabolism pathway.

We next analyzed the expression of iNOS and arginase-1 in PLT-BMDMs after the stimulation with zymosan and found that PLT-BMDMs expressed a lower level of iNOS and a higher level of arginase-1 in PLT-BMDMs as compared with control BMDMs (Fig 4C). These results suggest that PLT-sup similarly modulates the signaling for the expression of iNOS and arginase-1 mediated by distinct receptors for microbial components.

### PLT-sup modulated LPS-induced NF-κB signaling

**Fig 5:**
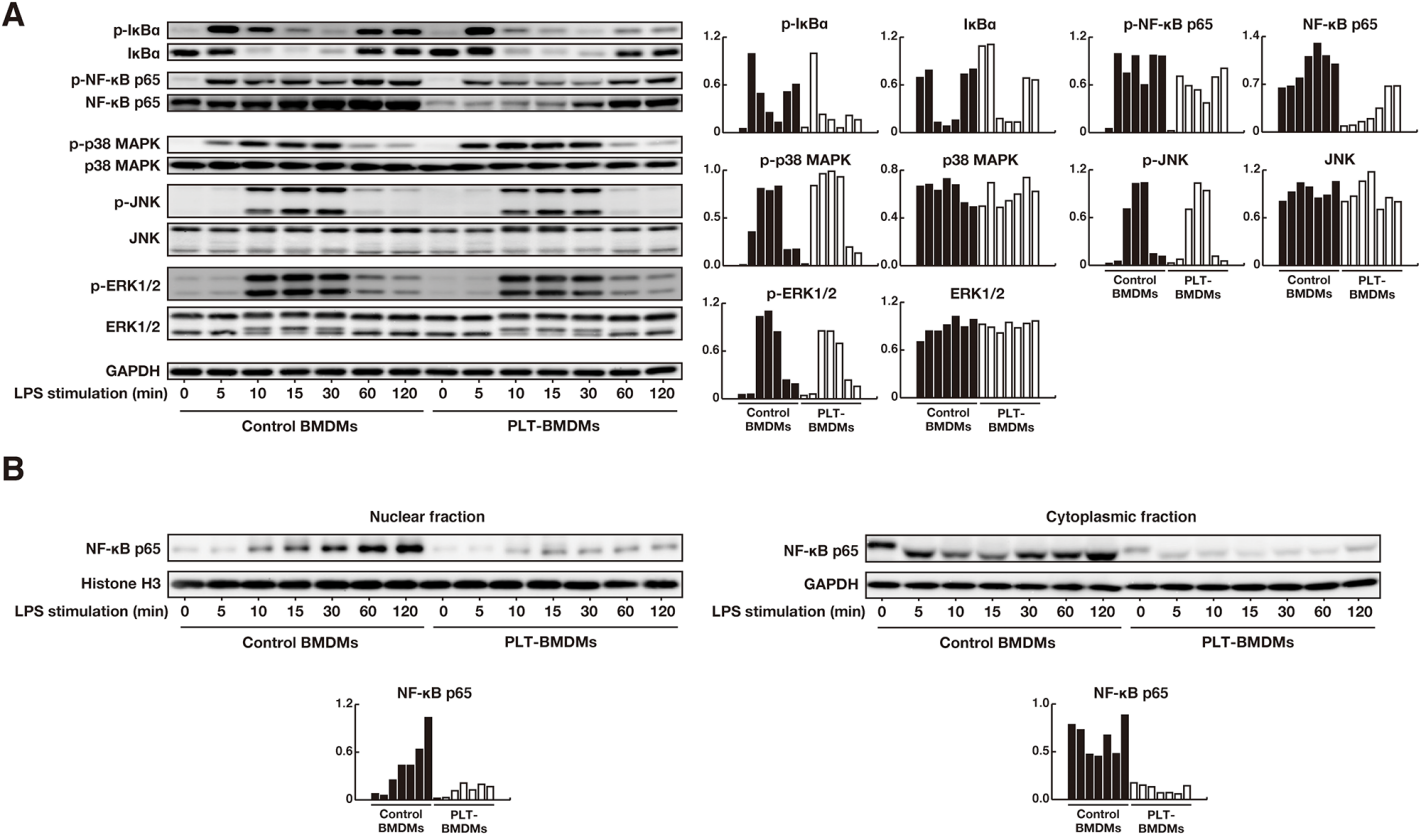
Western blotting analyses of NF-κB and MAPK signaling pathways in BMDMs after LPS stimulation. (A) PLT-BMDMs and control BMDMs (2.5 × 10^6^ cells) were stimulated with LPS (50 ng/mL) for 0–120 min. Cells were then lysed in 1 × SDS sample buffer, and the cell lysates were subjected to western blotting analysis with antibodies against phospho-IκBα, total IκBα, phospho-NF-κB p65, total NF-κB p65, phospho-p38 MAPK, total p38 MAPK, phospho-JNK, total JNK, phospho-ERK1/2, total ERK1/2 or GAPDH. The relative intensity of each band after normalization to the corresponding level of GAPDH is shown in the right panel. Experiments were repeated three times, and representative results are shown. (B) PLT-BMDMs and control BMDMs (2.5 × 10^6^ cells) that had been stimulated with LPS (50 ng/mL) for 0–120 min were separated into cytoplasmic and nuclear fractions. Each fraction was subjected to western blotting analysis with antibodies against NF-κB p65. GAPDH and histone H3 were used as controls for the cytoplasmic and nuclear fractions, respectively. The relative intensity of each band after normalization to the level of GAPDH or histone H3 is shown in the lower panel. Experiments were repeated three times, and representative results are shown.

To elucidate intracellular mechanisms underlying the reduced iNOS expression by PLT-sup, we assessed downstream signaling pathways of TLR4 in PLT-BMDMs. Since the nuclear factor-kappa B (NF-κB) signaling is thought to be the most important pathway involved in the regulation of iNOS expression [33, 34], we first focused on NF-κB p65 (a transcription factor of NF-κB signaling) and inhibitor of NF-κB (IκBα) (an inhibitor of nuclear translocation of NF-κB p65) [35]. PLT-BMDMs and control BMDMs were stimulated with LPS for 0–120 min, and the phosphorylation of IκBα and NF-κB p65 was detected by western blotting. The phosphorylation of IκBα in control BMDMs appeared to be biphasic; i.e., phosphorylated IκBα was increased at 5–10 min and 60–120 min after LPS-stimulation (Fig 5A). In PLT-BMDMs, however, phosphorylation of IκBα was suppressed particularly in the late phase. We also found that the total amounts of NF-κB p65 were decreased in PLT-BMDMs at all time periods examined (0–120 min). We then examined mitogen-activated protein kinase (MAPK) pathways mediated by ERK, JNK and p38 MAP kinase. The phosphorylation of ERK, JNK and p38 MAP kinase peaked 10–30 min after LPS-stimulation in PLT-BMDMs and control BMDMs, and we detected no substantial changes between PLT-BMDMs and control BMDMs in the kinetics of either the total amounts or the phosphorylation levels of these MAPK-related proteins.

Since phosphorylation of IκBα and total expression of NF-κB p65 were reduced in PLT-BMDMs, we next assessed the nuclear translocation of NF-κB p65. After PLT-BMDMs and control BMDMs were stimulated with LPS, the time-dependent changes in the amounts of NF-κB p65 in the cytoplasmic and nuclear fractions were detected by western blotting. NF-κB p65 in the nuclear fraction from control BMDMs was gradually increased from 5–120 min after stimulation, whereas NF-κB p65 from PLT-BMDMs remained at lower levels (Fig 5B). It is unclear, however, whether the reduced nuclear translocation of NF-κB p65 in PLT-BMDMs was due to the decreased expression of NF-κB p65 or the decreased phosphorylation of IκBα.

## Discussion

Nitric oxide produced by macrophages plays crucial roles in protection against microbial infection, but the excessive production of NO causes aggravation of LPS-induced septic shock [4, 9]. We previously reported that platelets attenuated LPS-induced macrophage inflammatory responses via soluble factor(s) [8]. In the present study, we focused on the intracellular processes leading to suppression of LPS-induced NO production by platelet-derived factors, and found a decrease of iNOS expression and an increase in arginase-1 expression, both of which are critical enzymes involved in NO synthesis and L-arginine metabolism (Figs 2 and 3). Arginase-1 is also known as a macrophage polarization marker molecule and is selectively expressed in M2-macrophages (AAMs). However, BMDMs cultured with PLT-sup appeared to be somewhat different from typical M2-macrophages because no significant increase in the expression of other markers such as *Fizz1*, *Ym1*, and *Mrc1* was detected (Fig 3A). Although M2-macrophages are known to suppress inflammatory responses through the production of anti-inflammatory cytokine IL-10, PLT-BMDMs have been shown to secrete an undetectable level of IL-10 (Ando *et al*., unpublished observation).

Macrophages expressing arginase-1 are distributed in various inflammatory tissues *in vivo* and can also be generated *in vitro* by culturing macrophages with IL-4, IL-13, and oxidized low-density lipoprotein (Ox-LDL) [21, 36]. However, so-called arginase-1-expressing macrophages are suggested to play an immunosuppressive role; e.g., the depletion of L-arginine from the microenvironment surrounding T cells by arginase-1-expressing cells results in the inhibition of antigen-specific proliferation and cytokine production [37, 38]. In the spleen, for example, red pulp macrophages are exposed by granule components spontaneously secreted from neighboring platelets, and macrophage functions might be negatively regulated so as not to exhibit an excessive response to bacterial components. Several recent reports, in turn, suggested that arginase-1-expressing macrophages played important roles in wound healing and tissue repairs [39, 40]. Under certain pathophysiological conditions, platelets adhere and aggregate at sites of vascular injury, and release various biologically active substances [41, 42], and adjacent macrophages are thought to differentiate into arginase-1-expressing macrophages and to function as immunoregulators. Because arginase-1 enhances collagen synthesis, cell proliferation and fibrosis through the generation of proline and polyamines [43–45], arginase-1-expressing macrophages at the sites of vascular injury may contribute to wound healing.

In this study, we observed suppression of the IκBα phosphorylation and decreased nuclear translocation of NF-κB p65 in LPS-stimulated PLT-BMDMs accompanied by decreased expression of iNOS (Fig 5). We previously reported that LPS-induced production of TNF-α and IL-6 by BMDMs were also suppressed by platelet-derived soluble factor(s) [8]. The inhibition of NF-κB signaling in PLT-BMDMs is likely to affect the expression of these cytokines as well as iNOS expression. Since the changes in arginase-1 and iNOS expression were also observed when PLT-BMDMs were stimulated with zymosan (Fig 4), a common activation signal from TLR2 and TLR4, such as a MyD88-dependent pathway, might be suppressed in PLT-BMDMs.

Chang and co-workers recently reported that a conditioned medium of a mouse hepatoma cell line induced arginase-1 expression in BMDMs in association with a decrease of NF-κB p65. They suggested that NF-κB p65 was rapidly degraded via p62/SQSTM1-mediated selective autophagy in a TLR2 signaling-dependent manner [46]. One possibility is that PLT-sup increased ariginase-1 expression and decreased NF-κB p65 expression in BMDMs through a similar process. On the other hand, Qualls and co-workers found that macrophages after mycobacterium infection expressed arginase-1 through the TLR pathway. The expression of arginase-1 has been shown to be mediated by cytokines such as IL-6, IL-10, and G-CSF in an autocrine/paracrine fashion [47]. It seems unlikely, however, that such a process is implicated in the arginase-1 expression in PLT-BMDMs, because we did not detect the potentiation of STAT3 phosphorylation essential for the arginase-1 expression (Ando *et al*., unpublished observation).

We previously reported that the attenuation of macrophage responses was mediated by platelet-derived heat-labile macromolecule(s), most likely protein(s) or protein-containing macromolecule conjugate(s) [8], and characterization of these factor(s) may provide an insight into the roles of platelets in the modulation of macrophage functions. The molecular mechanisms underlying the regulation of arginase-1 expression and NF-κB signaling by platelets should be elucidated in a future study. In conclusion, PLT-sup suppresses inflammatory responses of BMDMs via the negative regulation of NF-κB signaling, leading to lowered expression of iNOS and enhanced L-arginine catabolism by arginase-1. Further characterization of the platelet-dependent modulation of macrophages may contribute to our understanding of macrophage inflammatory responses and the development of novel therapeutic approaches for infectious diseases.

## Supporting Information

S1 Fig Cell surface expression of CD62P on thrombin-activated platelets. Washed platelets were suspended in ASF104 serum-free medium (1 × 10^8^ cells/mL) and incubated with or without 0.5 U/mL thrombin for 15 min at 37°C. The platelets were then treated with PE-conjugated anti-mouse CD62P antibody (REA344) (Miltenyi Biotec, Bergisch Gladbach, Germany) at 4°C for 15 min and analyzed by a flow cytometer (FACSVerse).
(PDF)

S2 Fig Attenuation of LPS-induced NO production from BMDMs by supernatants of platelets activated by various stimuli. Washed platelets were suspended in ASF104 serum-free medium (1 × 10^8^ cells/mL) and stimulated with 0.5 U/mL thrombin, 20 μM ADP or 2 μg/mL collagen (Nitta Gelatin, Osaka, Japan) for 15 min at 37°C. The supernatants were collected by centrifugation (800 × g, 15 min, 4°C) followed by filtration with a membrane (0.22 μm pore) (Thrombin-PLT-sup, ADP-PLT-sup and Collagen-PLT-sup). BMDMs (4 × 10^5^ cells) were cultured for 24 h with a platelet supernatant in a 24-well plate, and stimulated with complete medium containing LPS (50 ng/mL) for 24 h. The production of NO_2_^-^ in the culture supernatant was determined. BMDMs were also cultured with 0.5 U/mL thrombin, 20 μM ADP, 2 μg/mL collagen, or unstimulated platelet supernatant (Resting-PLT-sup). Experiments were performed in quintuplicate and repeated three times. The data are presented as the mean ± SEM. ***p < 0.005. Representative results from the three experiments are shown.
(PDF)

S3 Fig Effect of an arginase-1 inhibitor on LPS-induced NO production from PLT-BMDMs. BMDMs (4 × 10^5^ cells) were cultured for 24 h with PLT-sup in the presence or absence of 2S-amino-4-[[(hydroxyamino)iminomethyl]amino]-butanoic acid (nor-NOHA) (Cayman Chemical, MI, USA) in a 24-well plate, and stimulated with complete medium containing LPS (50 ng/mL) for 24 h. The production of NO_2_^-^ was determined. Experiments were performed in quintuplicate and repeated three times. The data are presented as the mean ± SEM. *p < 0.05, ***p < 0.005 vs. controls. Representative results from the three experiments are shown.
(PDF)
