# Supplementary figures and images for: Platelet Supernatant Suppresses LPS-Induced Nitric Oxide Production from Macrophages Accompanied by Inhibition of NF-κB Signaling and Increased Arginase-1 Expression
Source: PLoS One. 2016 Sep 2;11(9):e0162208. doi: 10.1371/journal.pone.0162208 (PMC5010197; doi:10.1371/journal.pone.0162208)

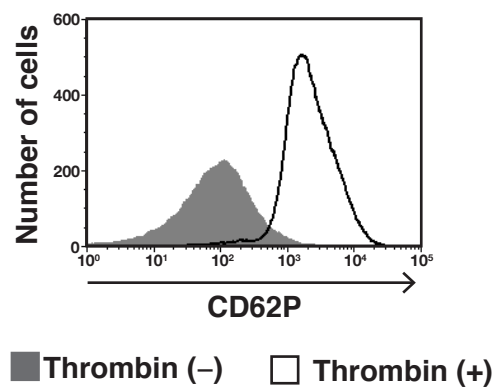

S1 Fig. Cell surface expression of CD62P on thrombin-activated platelets.

Supplement: S1 Fig — Washed platelets were suspended in ASF104 serum-free medium (1 × 10^8^ cells/mL) and incubated with or without 0.5 U/mL thrombin for 15 min at 37°C. The platelets were then treated with PE-conjugated anti-mouse CD62P antibody (REA344) (Miltenyi Biotec, Bergisch Gladbach, Germany) at 4°C for 15 min and analyzed by a flow cytometer (FACSVerse). (PDF) [file pone.0162208.s001.pdf]

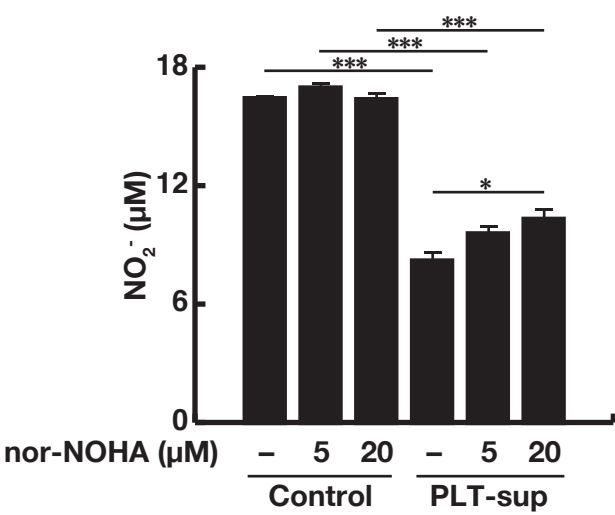

S3 Fig. Effect of an arginase-1 inhibitor on LPS-induced NO production from PLT-BMDMs.

Supplement: S3 Fig — BMDMs (4 × 10^5^ cells) were cultured for 24 h with PLT-sup in the presence or absence of 2S-amino-4-[[(hydroxyamino)iminomethyl]amino]-butanoic acid (nor-NOHA) (Cayman Chemical, MI, USA) in a 24-well plate, and stimulated with complete medium containing LPS (50 ng/mL) for 24 h. The production of NO2- was determined. Experiments were performed in quintuplicate and repeated three times. The data are presented as the mean ± SEM. *p < 0.05, ***p < 0.005 vs. controls. Representative results from the three experiments are shown. (PDF) [file pone.0162208.s003.pdf]
