# Supplementary material for: Platelet Supernatant Suppresses LPS-Induced Nitric Oxide Production from Macrophages Accompanied by Inhibition of NF-κB Signaling and Increased Arginase-1 Expression
Source: PLoS One. 2016 Sep 2;11(9):e0162208. doi: 10.1371/journal.pone.0162208 (PMC5010197; doi:10.1371/journal.pone.0162208)
Supplement: S2 Fig — Washed platelets were suspended in ASF104 serum-free medium (1 × 10^8^ cells/mL) and stimulated with 0.5 U/mL thrombin, 20 μM ADP or 2 μg/mL collagen (Nitta Gelatin, Osaka, Japan) for 15 min at 37°C. The supernatants were collected by centrifugation (800 × g, 15 min, 4°C) followed by filtration with a membrane (0.22 μm pore) (Thrombin-PLT-sup, ADP-PLT-sup and Collagen-PLT-sup). BMDMs (4 × 10^5^ cells) were cultured for 24 h with a platelet supernatant in a 24-well plate, and stimulated with complete medium containing LPS (50 ng/mL) for 24 h. The production of NO2- in the culture supernatant was determined. BMDMs were also cultured with 0.5 U/mL thrombin, 20 μM ADP, 2 μg/mL collagen, or unstimulated platelet supernatant (Resting-PLT-sup). Experiments were performed in quintuplicate and repeated three times. The data are presented as the mean ± SEM. ***p < 0.005. Representative results from the three experiments are shown. (PDF) [file pone.0162208.s002.pdf]

S2 Fig

Ando *et al*.

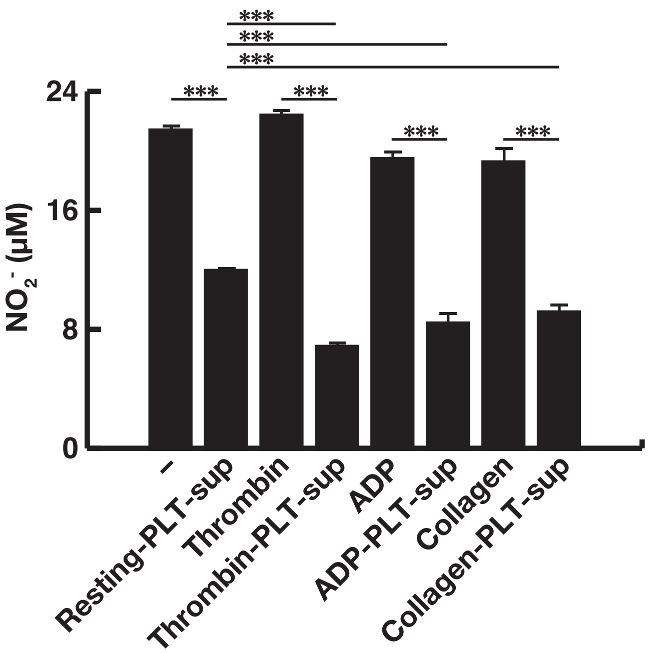

S2 Fig. Attenuation of LPS-induced NO production from BMDMs by supernatants of platelets activated by various stimuli.
